# Attention and Specificity in Unconscious Perceptual Learning

**DOI:** 10.1068/ig5

**Published:** 2013-10-01

**Authors:** D Carmel, M Carrasco

**Affiliations:** University of Edinburgh, UK; New York University, USA

## Abstract

Practicing perceptual tasks improves performance. This is known as perceptual learning (PL). Visual PL is highly specific to trained stimulus locations, indicating neural plasticity in early retinotopic visual regions. Attention facilitates PL, but would it also affect PL of stimuli that observers remain unaware of? Here, we first measured performance on a texture discrimination task used commonly in PL studies. In seven subsequent training sessions, similar textures were presented monocularly and suppressed from awareness by continuous flash suppression, where monocular stimuli are rendered invisible by dynamic displays presented to the other eye. Observers performed an attentional task on dominant-eye stimuli; texture discrimination targets were presented at attended and unattended locations in the suppressed eye. In a final session, we assessed texture discrimination again. We expected PL (improved texture discrimination) to be greatest at locations that were attended during training. Surprisingly, we found significant PL at both attended and unattended locations. Control experiments ruled out the possibility that improvements were due to either repeated testing or training on the dominant eye's task. As observers were unaware of the texture stimuli during training, this finding indicates that attention can facilitate PL without awareness, and furthermore, can generalize it to untrained locations.

